# Generalization of the normal-exponential model: exploration of a more accurate parametrisation for the signal distribution on Illumina BeadArrays

**DOI:** 10.1186/1471-2105-13-329

**Published:** 2012-12-11

**Authors:** Sandra Plancade, Yves Rozenholc, Eiliv Lund

**Affiliations:** 1 Department of Community Medicine, Faculty of Health Sciences, University of Tromsø, 9037 Tromsø, Norway; 2 Department of Applied Mathematics, MAP5, 45 rue des Saints-Pères, University Paris Descartes, 75006 Paris, France

## Abstract

**Background:**

Illumina BeadArray technology includes non specific negative control features that allow a precise estimation of the background noise. As an alternative to the background subtraction proposed in BeadStudio which leads to an important loss of information by generating negative values, a background correction method modeling the observed intensities as the sum of the exponentially distributed signal and normally distributed noise has been developed. Nevertheless, Wang and Ye (2012) display a kernel-based estimator of the signal distribution on Illumina BeadArrays and suggest that a gamma distribution would represent a better modeling of the signal density. Hence, the normal-exponential modeling may not be appropriate for Illumina data and background corrections derived from this model may lead to wrong estimation.

**Results:**

We propose a more flexible modeling based on a gamma distributed signal and a normal distributed background noise and develop the associated background correction, implemented in the R-package NormalGamma. Our model proves to be markedly more accurate to model Illumina BeadArrays: on the one hand, it is shown on two types of Illumina BeadChips that this model offers a more correct fit of the observed intensities. On the other hand, the comparison of the operating characteristics of several background correction procedures on spike-in and on normal-gamma simulated data shows high similarities, reinforcing the validation of the normal-gamma modeling. The performance of the background corrections based on the normal-gamma and normal-exponential models are compared on two dilution data sets, through testing procedures which represent various experimental designs. Surprisingly, we observe that the implementation of a more accurate parametrisation in the model-based background correction does not increase the sensitivity. These results may be explained by the operating characteristics of the estimators: the normal-gamma background correction offers an improvement in terms of bias, but at the cost of a loss in precision.

**Conclusions:**

This paper addresses the lack of fit of the usual normal-exponential model by proposing a more flexible parametrisation of the signal distribution as well as the associated background correction. This new model proves to be considerably more accurate for Illumina microarrays, but the improvement in terms of modeling does not lead to a higher sensitivity in differential analysis. Nevertheless, this realistic modeling makes way for future investigations, in particular to examine the characteristics of pre-processing strategies.

## Background

Illumina BeadArray platform is a microarray technology offering highly replicable measurements of gene expression in a biological sample. Each probe is measured on average of thirty to sixty beads randomly distributed on the surface of the array, avoiding spatial artifacts and the reported probe intensity is the robust mean of the bead measurements. Fluorescence intensity measured on each bead is subject to several sources of noise (non-specific binding, optical noise, …). Thus the intensities produced by the microarray require a background correction in order to account measurement error. For that purpose, Illumina microarray design includes a set of non specific negative control probes which provides an estimate of the background noise distribution.

In genome-wide microarrays, the observed intensity of a probe is usually modeled as the sum of a signal and a background noise. Namely, let *X* be the observed intensity of a given probe, we assume that

(1) X=S+B

where *S* is the true signal which counts for the abundance of the probe complementary sequence in the target sample and is independent of the background noise *B*. Only *X* is observed but the quantity of interest is the signal *S*. Therefore, a background correction adjusting the effect of noise on the true signal is necessary to enhance the biological validity of the results. In this context the knowledge of both signal and noise distributions provides a background correction procedure: the signal *S* is estimated by the conditional expectation of *S* given the observation *X*=*x* and given the distributions of *B* and *S*. Under parametric assumptions on *B* and *S*, the problem is limited to the estimation of the parameters. Besides, in many experimental contexts involving measurement error the normal distribution of the noise is assumed. Specific arguments for microarray data find their origins in analytical chemistry (see e.g. [1]).

Background correction of Affymetrix and two-color microarray data has been widely developed in literature (see [2] for a review and a comparison). Irizarry *et al*[3] proposed a parametric model for Affymetrix based on a exponential distribution of the signal, called normexp model. Several estimation procedures have been developed for this model. The first parameter estimation, still popular today, is the Robust Multi-array Average (RMA) procedure. Maximum Likelihood Estimation (MLE), incorporating the negative controls, has been later proposed and is considered to be more sensitive to the true parameter values (see [4]). These procedures can be found in Bioconductor^a^ packages including limma[5].

Illumina design differs from those of Affymetrix and two-color microarrays by including a set of negative probes which do not specifically target any regular probe. Aside from non specific hybridization, these negative probes do not hybridize and then have signals close to zero. Thus their observed intensity is *X*=*B.* As all probes from a given array correspond to the same biological sample and are subject to the same technical steps during the analysis process, the noise is generally assumed identically distributed on an array and the negative probes provide a sample from its distribution.

The background correction implemented in Illumina BeadStudio software is the subtraction of the estimated mean of the negative probe distribution. However, it creates a large amount of probes with negative intensities unusable in further analysis. The deletion of these probes is considered in some studies as an opportunity to gain statistical power when the number of strongly differentially expressed genes is large, but it can lead to an important loss of information. Ding *et al*[6] illustrate this phenomenon in their mice leukemia study: a large amount of corrected values are negative only in one group suggesting that the corresponding probes have discriminating ability. This issue is confirmed by Dunning *et al*[7] on spike-in data.

To avoid this problem, parametric models have been used on Illumina data with parameter estimations taking into account the specific design of Illumina microarrays. In this context, the normexp model has been first adapted. Ding *et al*[6] use the Maximum Likelihood Estimation (MLE) based on a Monte-Carlo Markov chain approximation and compare their method to an Illumina-adapted RMA procedure using an *ad hoc* rule of thumbs to estimate the parameters. Xie *et al*[8] go into details in normexp method comparison on experimental and simulated data. Lin *et al*[9] present a variance stabilizing transformation (VST) on a model involving both additive and multiplicative noises, which simultaneously denoise and transform the data. Replacing the classical *log*-transformation, VST produces less directly interpretable results and tends to produces very small fold changes, as underlined by Shi *et al*[10] who propose an original approach to compare methods offering different bias-precision trade-off by aligning the innate offset generated by each pre-processing strategy. They conclude in favor of the normexp model with robust ’non-parametric’ parameters associated to a quantile-normalization using control and regular probes. Besides, Chen *et al*[11] propose a gamma parametrisation of the background noise distribution to handle with the departure from normality observed on negative probes, associated with an exponential distribution of the signal. They emphasize that gamma-exponential model can provide an improvement in terms of differential analysis, but might not be an adequate parametrisation in some cases.

The spread of each background correction among the Illumina users is hard to evaluate since many authors do not mention precisely the pre-processing steps performed in their study. Nevertheless, the normexp model that will be especially examined in this paper is included in several widely used packages such as lumi and limma of Bioconductor^a^.

Despite its popularity the normexp model does not properly fit Illumina microarray data. This issue was raised by Wang and Ye [12], who estimate the density of the signal on an Illumina microarray with a kernel-based deconvolution procedure. The shape of the estimated signal density does not present the characteristics of an exponential distribution and a gamma modeling seems more appropriate. We confirm these findings by implementing the kernel-based estimator by Wang and Wang [13] available in the R package decon. (The results are displayed in Additional file 1, Section 1). The signal density estimate exhibits a heavy tail which can not be fitted by an exponential distribution density. Nevertheless, kernel-based density estimators does not appear efficient to recover breakpoints in the density, and presents instabilities, which limits the interpretation of the signal density estimate in the microarray context. In this paper, we emphasize that the normal-exponential model is not flexible enough to model the signal-noise decomposition on Illumina microarrays by showing that the distance between the reconstructed density from the estimated parameters and the distribution of the observed intensities is large.

We propose an alternative model thereafter called “*normal-gamma* model” which addresses this lack of fit. In our model, the normal noise distribution is assumed and the signal on one array is assumed to be gamma distributed. As the exponential distribution is a special case of the gamma distribution, this model extends the normexp model. The potential of such generalization was already suggested by Xi *et al*[8] in their discussion. We derive the necessary estimation procedure by likelihood maximization. The good quality of fit is attested on two types of Illumina microarrays. The associated background correction is compared to methods based on the normexp model in terms of quality of estimation of the signal and checked for robustness on simulated data. The characteristics of the background correction procedures are compared on a set of spike-in data, and a parallel is drawn with the same characteristics studied on normal-gamma simulated data. Finally, the normexp and normal-gamma background corrections are compared on two dilution data sets.

The paper is organized as follows. The experimental and simulated data sets as well as the estimation procedures are presented in Section “Methods”: the notations and the general model-based background correction formula are gathered in Section “General model-based background correction formula”; the previous models developed for Illumina microarray background correction, including the normexp model, are summarized in Section “Previous modelings”; Section “A new modeling: the normal-gamma model” presents the proposed alternative parametric model built with normal noise and gamma distributed signal, as well as a parametric estimation procedure and its associated background correction. The performances of this new model are evaluated on simulated, spike-in and dilution data sets in Section “Results and discussion”. The impact of this more flexible parametrisation on background correction as well as the perspectives for further pre-processing analyses are discussed in Section “Conclusions”. The normal-gamma parameter estimation and the associated background correction are implemented in the R-package NormalGamma. The scripts used to produce the tables and figures are available in Additional file 2.

## Methods

### Materials

#### Experimental data sets

● (*E*_1_)**Nowac data**[14]. The gene expression profile in peripheral blood from ten controls in the Norwegian Woman And Cancer study has been analysed on Human HT-6 v4 Expression BeadChips. The whole probe set including 48,000 bead types has been considered, as well as a restricted set of 25,519 bead types according to Illumina annotation files. Details on laboratory experiments are given in Additional file 1: Section 2.1. The data are provided in Additional file 3.

● (*E*_2_)**Leukemia mice data**[6]. Total RNA from samples of spleen cells from four mice have been analysed on Mouse-6 v1 BeadChips. Experiment description and data are available in [6]

● (*E*_3_)**Spike-in data**[15]. HumanWG-6 v2 BeadChips have been customized to include 34 bead types, refered as ’spikes’, whose corresponding target sequence is absent from the human genome in addition to the 48,000 regular probes. The 34 spikes were introduced at 12 different concentrations (0pm, 0.01pm, 0.03pm, 0.1pm, 0.3pm, 1pm, 3pm, 10pm, 30pm, 100pm, 300pm, 1000pm) in a human biological sample. Each sample corresponding to a spike concentration has been analysed on four arrays. The data are available at http://rafalab.jhsph.edu/.

● (*E*_4_)**MAQC data**[16]. Two pure samples, Universal Reference RNA (HBRR) and Human Brain Reference RNA (UHRR) were mixed in four different proportions (100%/0%, 75%/25%, 25%/75%, 0%/100%). Five replications of each sample have been analysed on HumanWG-6 v1 BeadChips. The data are available on GEO (access number GSE5350).

● (*E*_5_)**Dilution data**[17]. The pure samples UHRR and HBRR were mixed at different proportions (100%/0%, 99%/1%, 95%/5%, 90%/10%, 75%/25%, 50%/50%, 25%/75%, 10%/90%, 0%/100%). Each mixed sample has been analysed with four different starting RNA quantities (250ng, 100ng, 50ng, 10ng). Six HumanWG-6 v3 BeadChips were used.

#### Simulated data sets

For each data set, *N*=100 random arrays including a vector **X**^*ℓ*^of length *n*_reg_=25000 corresponding to the regular probe intensities and a vector **X**^0,*ℓ*^of length *n*_neg_=1000 corresponding to the negative probe intensities are generated. The values of the nine parameter sets as well as the details of the simulations are given in Additional file 1: Section 2.2 

● (*S*_1_)**Normal-gamma and normexp models**. For each repetition *ℓ*=1,…,*N*, **X**^*ℓ*^is generated as the sum of a gamma and a normal-distributed sample, and **X**^0,*ℓ*^is drawn from a normal distribution. Six sets of parameters are computed from two microarrays in data sets (*E*_1_) and (*E*_2_), based on normexp and normal-gamma models in order to get realistic values (sets 1-6). The normexp parameters are actually degenerated normal-gamma parameters where the shape is equal to 1.

● (*S*_2_)**Mixture noise distribution**. A mixture of normal and *χ*^2^distributions with different proportions (0, 0.1, 0.25, 0.5, 0.75,1) is considered for the background noise. These distributions model a departure from normality with a heavier right tail for larger values of *p*. The mixture densities are presented in Additional file 1: Section 5. The signal is generated from a gamma distribution with parameter values from set 1.

● (*S*_3_)**Replicates**. We mimic replicate measurements from a biological sample by simulating *N* arrays with the same signal sample generated from a gamma distribution. The background noise and negative probe intensities are independently drawn from a normal distribution for each array. The values of the parameters are computed from the first array in (*E*_3_) (set 7). Replicates from the normal-exponential model are drawn in the same way with parameter values estimated on the same array with two normexp estimates (sets 8 and 9).

● (*S*_4_)**Replicates with empirical background noise**. Similarly to (*S*_3_), the signal drawn from a gamma distribution with parameter values from set 7 is identical on each array. In order to get a realistic noise distribution, the negative probe and background noise intensities are sampled from the global set of quantile-normalised negative probe intensities measured in the experimental data set (*E*_3_).

### General model-based background correction formula

#### Notations

Throughout this article, the background correction is processed on one single array corresponding to one biological sample. For a given probe *j*, we denote by *X*_*j*_ the observed intensity, *S*_*j*_ the non-observable underlying signal and *B*_*j*_ its background noise. For a negative control probe, *S*_*j*_is assumed to be 0. Let *J* and *J*_0_be respectively the index of regular and negative probes on the array. We denote by *f*_*X*_, *f*_*S*_ and *f*_*B*_the densities of respectively the observed intensity, the unknown signal of interest and the background noise.

We denote by $f_{\mu ,\sigma^{2}}^{\text{norm}}$ the density of the normal distribution with mean *μ*and variance *σ*^2^and by *ϕ*and *Φ* the density and cumulative distribution function of the normal distribution with mean 0 and variance 1. We denote by $f_{\alpha }^{\text{exp}}=(1/\alpha )\exp(-x/\alpha )$ the density of the exponential distribution with mean *α* and $f_{\theta ,k}^{\text{gam}}=x^{k-1}\exp\left(-x/\theta \right)/(\theta^{k}\Gamma (k))$ the density of the gamma distribution with scale parameter *θ*and shape parameter *k*. The exponential distribution is a special case with *k*=1 and *θ*=*α*.

Given a parametric density and a procedure of estimation of its parameters, we call *plug-in* density, this density for the estimated parameters.

#### Model-based background correction

The model based background correction (BgC) incorporates information from both signal and noise distributions. Under the additive model (1) assuming independence of *S* and *B*, *f*_*X*_ is the convolution product of *f*_*S*_and *f*_*B*_. For an observed probe intensity *x*, the signal *S* is estimated by the conditional expectation of *S* given the observation *X*=*x* and the densities *f*_*B*_and *f*_*S*_(more details can be found in [8]):

(2) $$\begin{array}{lcrr} \hat{S}(x) & = & E\left[S|X=x,f_{B},f_{S}\right]=\int s\frac{f_{S}(s)f_{B}(x-s)}{f_{X}(x)}\mathrm{ds} & \\ & = & \int sf_{S}(s)f_{B}(x-s)\mathrm{ds}/\int f_{S}(s)f_{B}(x-s)\mathrm{ds.}\; \end{array}$$

### Previous modelings

#### The normal model for negative probes

The design of Illumina BeadArrays provides a sample of the background distribution through the negative probes. We have compared the density histogram of negative probes to the *plug-in* normal density $f_{\hat{\mu },\hat{\sigma }}^{\text{norm}}$ obtained by using robust estimators of the parameters on data sets (*E*_1_) and (*E*_2_).

The results of this comparison are presented in Additional file 1: Section 3.1. As expected, the empirical distribution is essentially normal but we notice a slightly heavier right tail. It may be interpreted as intensities of wrongly designed negative probes which partially hybridize with some material present in the biological sample.

#### The normal-exponential model

The normexp model is a parametric model for the noise-signal decomposition on one array. We recall it briefly and refer for example to [8] for more details. For every probe *j*,

$$\begin{array}{l} X_{j}=S_{j}+B_{j},\\ S_{j}∼\left\{\begin{array}{ll} \text{Exp}(\alpha ), & \text{if}\;j\in J,\\ 0 & \text{if}\;j\in J_{0}, \end{array}\right)\\ B_{j}∼N(\mu ,\sigma^{2}),\\ S_{j}⊥B_{j}, \end{array}$$

where ⊥ denotes the independence between variables. The parameters (*μσα*) depend on the given array.

For computational reason, the *X*_*j*_’s are usually and often implicitly assumed to be independent. The existence of pathways between genes violates this assumption. Nevertheless, as a small proportion of genes are involved, results are reliable. According to the convolution structure (see Section “Model-based background correction”), the density of the *X*_*j*_’s is:

(3) $$f_{\mu ,\sigma ,\alpha }^{\text{nexp}}(x)=\frac{1}{\alpha }\exp\left(\frac{\sigma^{2}}{2\alpha^{2}}-\frac{x-\mu }{\alpha }\right)\Phi \left(\bar{x}\right),$$

where $\bar{x}=(x-\mu -\sigma^{2}/\alpha )/\sigma$. Denoting *Θ* = (*μ*,*σ*,*α*), from (2), the background corrected intensity for an observed intensity *x* is:

(4) $${\hat{S}}^{\text{nexp}}(x|\Theta )=\sigma \left(\bar{x}+\frac{\phi (\bar{x})}{\Phi (\bar{x})}\right).$$

#### Normal-exponential model fit

We consider the data sets (*E*_1_) and (*E*_2_). For each array, the following procedure is implemented: 

● Computation of the estimators $\left(\hat{\mu },\hat{\sigma },\hat{\alpha }\right)$ of the normexp model with the methods described in [8]: 

1. Maximum Likelihood Estimation (MLE) using both regular and negative probes,

2. Robust Multiarray Analysis (RMA) adapted from Affymetrix method,

3. NP estimation obtained by the method of moments applied to negative and regular probes,

4. Bayesian estimation. Note that the bayesian estimation results are not presented as they are nearly identical to MLE, as pointed out by Xie *et al*[8].

● For each parameter estimation method, plot of the *plug-in* density $f_{\hat{\mu },\hat{\sigma },\hat{\alpha }}^{\text{nexp}}$.

● Plot of an irregular density histogram of all regular probe intensities of the array using the R-package histogram available on the CRAN with default irregular setting (see [18]). Even though adaptive irregular histograms are not commonly used to describe microarray data, they have been proved to offer a better approximation in a general framework. Moreover, they appear especially relevant to estimate microarray distributions which present high irregularities.

**Figure 1:**
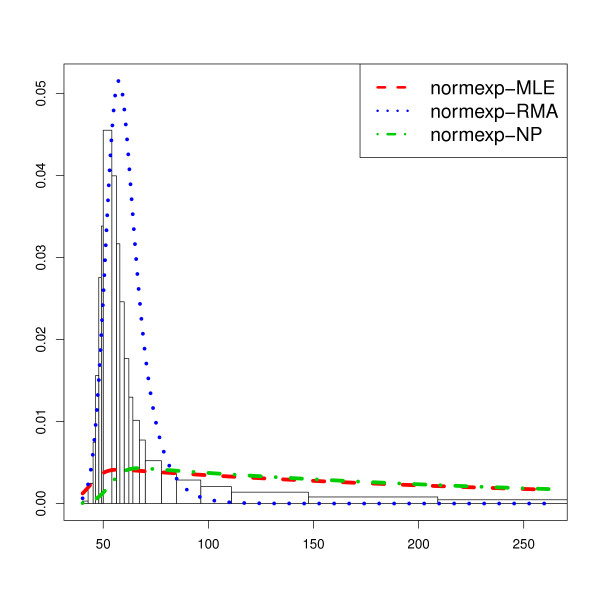
**Normal-exponential fit.** Normal-Exponential estimation for one array from (*E*_1_) after removal of imperfectly designed probes: irregular density histogram of all regular probe intensities and the *plug-in* normexp density of the regular probes with MLE, RMA and NP parameter estimates.

Figure 1 shows the results for this procedure on one array from (*E*_1_) after removal of imperfectly designed probes (more arrays are presented in Additional file 1: Section 3.2). Apart from the RMA method, the estimated density does not fit the density histogram and even the RMA estimator is not satisfying from a statistical point of view. One can remark that RMA underestimates the high expressions while the other methods tend to overestimate their contributions.

Besides Xie *et al*[8] show that the MLE and the NP estimation provide satisfying estimators of the parameters on normexp simulated data. Thus the difference between the histogram of the observed intensities and the plug-in density does not come from a poor estimation of the parameters but results from an unsuitable parametric model.

### A new modeling: the normal-gamma model

The poor fitting of the normexp model shown above, as well as the preliminary observations based on non-parametric estimation procedures, call for a more suitable parametric model for Illumina BeadArrays. According to Section “Previous modelings” the normal assumption for the negative probes appears relevant. We consider the gamma distribution as an extension of the exponential distribution to model the signal intensities. Besides, as a scale mixture of exponential distributions (see [19]), the gamma distribution is a natural generalization which helps to take into account different probe hybridization behaviors which could count for different exponential life times. This defines a more flexible parametric model called the *normal-gamma* model that we propose to apply to Illumina BeadArrays.

#### The normal-gamma model

The normal-gamma model is defined as follows. For every probe *j*:

(5) $$\begin{array}{ll} X_{j}=S_{j}+B_{j}, & \\ S_{j}∼\left\{\begin{array}{ll} \Gamma (\theta ,k), & \text{if}\;j\in J,\\ 0 & \text{if}\;j\in J_{0}, \end{array}\right)\\ B_{j}∼N(\mu ,\sigma^{2}),\\ S_{j}⊥B_{j}. \end{array}$$

The parameters (*μ*,*σ*,*k*,*θ*) depend on the given array. This model offers more flexibility than the normexp model but requires the estimation of one more parameter.

According to the convolution structure (see Section “Model-based background correction”), the density of *X*_*j*_ is the convolution product of the densities of *S*_*j*_and *B*_*j*_, namely:

(6) $$f_{\mu ,\sigma ,k,\theta }^{\text{ng}}(x)=\int \overset{\text{gam}}{\underset{k,\theta }{f}}(t)f_{\mu ,\sigma }^{\text{norm}}(x-t)\mathrm{dt.}$$

This density does not have any analytic expression as the normexp density (3). Nevertheless, good and fast numerical approximations can be computed using the Fast Fourier Transform (fft) and tail approximations to ensure stability. Our implementation based on fft is detailed in Additional file 1: Section 7.

#### Parameter estimation in the normal-gamma model

The parameters (*μ*,*σ*,*k*,*θ*) of the normal-gamma distribution are estimated by the Maximum Likelihood Estimator (MLE):

(7) $$(\hat{\mu },\hat{\sigma },\hat{k},\hat{\theta })=\arg\;\underset{\left(\mu ,\sigma ,k,\theta \right)}{\max}L\left((\mu ,\sigma ,k,\theta )|\text{X},{\text{X}}^{0}\right)$$

where

$$L\left((\mu ,\sigma ,k,\theta )|\text{X},{\text{X}}^{0}\right)=\underset{j\in J}{\prod }f_{\mu ,\sigma ,k,\theta }^{\text{ng}}(X_{j})\cdot \underset{j\in J_{0}}{\prod }f_{\mu ,\sigma }^{\text{norm}}(X_{j})$$

is the likelihood from the two sets of observations **X**={*X*_*j*_,*j*∈*J*} and **X**^0^={*X*_*j*_,*j*∈*J*_0_} measured on regular and negative probes, respectively. Thanks to the fft-based approximation of $f_{\mu ,\sigma ,k,\theta }^{\text{ng}}$ the maximum likelihood estimation can be numerically computed using classical minimization algorithms (see Additional file 1: Section 7).

#### Background corrected intensity for the normal-gamma model

Denoting now *Θ* = (*μ*,*σ*,*k*,*θ*), we derive from (2) the back- ground corrected intensity for an observed intensity *x*:

(8) $$\begin{array}{ll} {\hat{S}}^{\text{ng}}(x|\Theta ) & =\int s\overset{\text{gam}}{\underset{k,\theta }{f}}(s)f_{\mu ,\sigma }^{\text{norm}}(x-s)\;\mathrm{ds}/f_{\mu ,\sigma ,k,\theta }^{\text{ng}}(x))\\ & =\mathrm{k\theta}f_{\mu ,\sigma ,k+1,\theta }^{\text{ng}}(x)/f_{\mu ,\sigma ,k,\theta }^{\text{ng}}(x)) \end{array}$$

using the equality $\frac{}{}sf_{k,\theta }^{\text{gam}}(s)=\mathrm{k\theta}f_{k+1,\theta }^{\text{gam}}(s)$ valid for every *s*>0. This formula allows fft-based computations for the background correction.

#### Inference of negative probes from Illumina detection p-values

Most publicly available data sets do not present the negative probe intensities. Nevertheless, for each regular probe, Illumina provides a detection p-value equal to the proportion of negative probes which have intensities greater than that probe on a given array. Following the idea from Shi *et al*[20] we propose to infer the negative probe intensities from the detection p-values (see details in Additional file 1: Section 8.1). For the normexp and the normal-gamma models, the estimates of the parameters and reconstructed signals obtained with the true and inferred negative probe intensities are compared on the ten arrays from (*E*_1_). We observe that the error resulting from inference of the negative probe is negligible, with a relative error of order 10^−3^ to 10^−4^ on parameter estimation and 10^−4^to 10^−5^ on signal estimation (see Additional file 1: Section 8.2).

## Results and discussion

### Fit on Illumina BeadArray data

**Figure 2:**
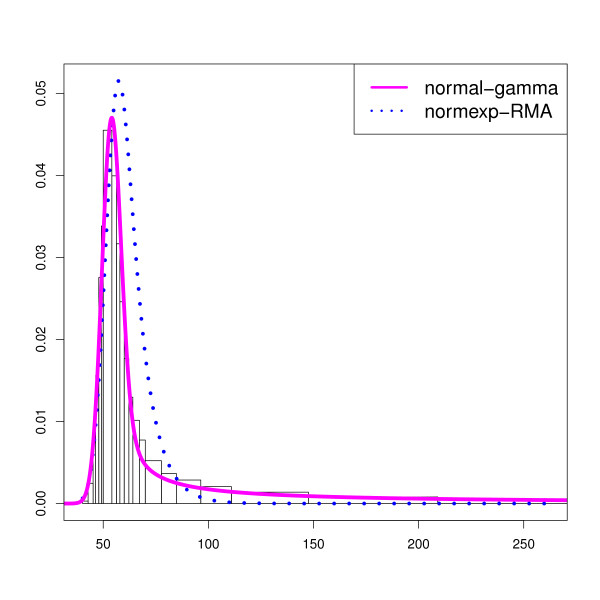
**Normal-exponential and normal-gamma fit.** Normal-Gamma estimation for one array from (*E*_1_) after removal of imperfectly designed probes: irregular density histogram of all regular probe intensities, *plug-in* normexp density with RMA estimate and *plug-in* normal-gamma density with MLE estimate.

Similarly to Section “Previous modelings”, we compare the irregular density histogram of the regular probe intensities with the *plug-in* normexp densities using RMA, MLE, NP methods and the *plug-in* normal-gamma density using a Maximum Likelihood Estimate of (*μ*,*σ*,*k*,*θ*) on the data sets (*E*_1_) and (*E*_2_). The results, similar along the arrays, are illustrated in Figure 2 on one array from (*E*_1_) (more plots are presented in Additional file 1: Section 3.2). We do not add the MLE and NP *plug-in* density estimates for the normexp model which have already been shown not to fit the data.

Thanks to the larger flexibility of the normal-gamma model, we observe that the distance between the MLE *plug-in* normal-gamma density and the histogram of the intensities is smaller than the corresponding distance using the normexp model with any estimation procedure. This graphical result is confirmed numerically using the *L*_1_-distance between the histogram and the reconstructed density defined by

(9) $$\ell_{1}(\hat{f},ĥ)=\int |\hat{f}(x)-ĥ(x)|\;\mathrm{dx},$$

where $\hat{f}$ represents one *plug-in* density estimate using either the normexp model or the normal-gamma model and ĥ represents the irregular density histogram obtained with the R-package histogram. Table 1 presents the mean of the relative deviation for the normexp estimators with respect to the deviation for the normal-gamma estimator:

$$\text{mean}\left(\frac{\ell_{1}(\hat{f_{i}},\hat{h_{i}})}{\ell_{1}({\hat{f}}_{i}^{\text{ng}},\hat{h_{i}})}\right)$$

where $\hat{f_{i}}$ is a normexp estimator of the regular probes density, and ${\hat{f}}_{i}^{\text{ng}}$ is the normal-gamma estimator for individual *i*. The mean is computed over the ten arrays from (*E*_1_) (with and without the non specific binding probes) and over the four arrays from (*E*_2_).

**Table 1:** Deviation between reconstructed intensities and observation histogram

	**Human**	**Human**	**Mice**
	**(all probes)**	**(remove bad probes)**	
nexp MLE	7.09	5.14	4.83
nexp RMA	2.96	3.18	2.71
nexp NP	7.69	5.50	5.29
Abs Dev normgam	0.17	0.21	0.20

Average deviation between normexp reconstructed density and histogram divided by the deviation between normal-gamma reconstructed density and histogram (First row: RMA estimator; second row: MLE normexp estimator; third row: NP normexp estimator). The fourth row gives the mean deviation of the normal-gamma estimator as a reference. The mean is computed over the ten arrays from (*E*_1_) with (first column) or without (second column) the non specific binding probes and over the four arrays from (*E*_2_) (third column).

The mean absolute deviation is 3 times smaller in favor of the normal-gamma density with respect to the normexp density using the RMA estimate, and 4 to 8 with respect to the normexp model using the MLE or NP estimates.

### Quality of estimation on simulated data

The quality of estimation of the normal-gamma model is assessed on the simulation data set (*S*_1_). The first two sets of parameters are non degenerate normal-gamma parameters, more realistic for modeling Illumina microarrays as shown in Section “Fit on Illumina BeadArray data”. They are used to evaluate the MLE normal-gamma parameter estimation and validate the associated background correction, and to quantify the improvement brought by the new normal-gamma background correction. The last four sets are actually degenerate normal-gamma parameters where the shape parameter *k* is set to 1, corresponding to normexp data, which enables to assess the potential loss of precision in parameter estimation brought by a more flexible modeling.

#### Parameter estimation

For each repetition *ℓ*=1,…,*N* we compute the normal-gamma MLE $\left({\hat{\mu }}^{\ell },{\hat{\sigma }}^{\ell },{\hat{k}}^{\ell },{\hat{\theta }}^{\ell }\right)$ of the parameters. Table 2 presents the relative *L*_1_-error for each parameter *β*∈{*μ*,*σ*,*k*,*θ*}:

$$\frac{1}{N}\overset{N}{\underset{\ell =1}{\sum }}\left|\beta -{\hat{\beta }}^{\ell }\right|/\mathrm{\beta .})$$

**Table 2:** **Relative*L***_**1**_**-error for each parameter in the normal-gamma model using MLE estimates**

	***μ***	***σ***	***k***	***θ***
set 1	7.1e-4	5.6e-3	9.3e-3	1.7e-2
set 2	1.3e-3	5.5e-3	1.0e-2	1.8e-2
set 3	3.5e-3	1.6e-2	6.9e-3	8.3e-3
set 4	4.5e-3	1.3e-2	8.9e-3	9.8e-3
set 5	2.1e-3	7.6e-3	2.6e-2	1.7e-2
set 6	3.5e-3	7.2e-3	3.9e-2	2.4e-2

The parameter estimation is of excellent quality for the gaussian distribution and of good quality for the gamma distribution.

To check wether the introduction of a fourth parameter in our model leads to a loss of precision in the parameter estimation, we compare the relative *L*_1_errors of the MLE parameter estimation in the normal-gamma and normexp models using the parameter sets 3 to 6, corresponding to normexp data. The results summarized in Table 3 indicate that the quality is unchanged for the variance parameter and that we pay a price of order 2 for *μ* and *θ*. Nevertheless, since the relative errors in these cases have order 10^−2^, this loss is negligible.

**Table 3:** Error in parameter estimation

	***μ***	***sigma***	***theta***
set 3	1.1	1.0	1.6
set 4	1.2	1.0	1.8
set 5	2.1	1.0	2.3
set 6	1.9	1.0	1.9

Ratio between the relative *L*_1_errors of the MLE estimation in the normal-gamma and in the normexp models for (*μ*,*σ*,*θ*) from normexp data.

#### Background corrected intensity

We now study the performance of the normal-gamma background correction (BgC) obtained in (8) with respect to the existing BgC methods in terms of quality of estimation of the signal on the simulated data set (*S*_1_). We compare the following BgC methods, detailed in [8]: 

0. Normal-gamma BgC in (8) with true parameters,

1. Normal-gamma BgC in (8) with MLE parameters,

2. Normal-exponential BgC in (4) with MLE parameters (referred to as normexp-MLE),

3. Normal-exponential BgC in (4) with RMA parameters (referred to as normexp-RMA),

4. Normal-exponential BgC in (4) with NP parameters (referred to as normexp-NP),

5. Background subtraction:

$${\hat{S}}^{\text{sub}}(x)=\max\left(x-\text{median}\{X_{j},j\in J_{0}\},0\right).$$

These methods are further denoted by ${\hat{S}}^{\left(i\right)}$ for *i*=0,…,5. For methods 1 to 4, the BgC is a two-step procedure: the parameters are estimated and then plugged respectively into (8) for method 1, and into (4) for methods 2 to 4. From a practical point of view, as the parameters are unknown, ${\hat{S}}^{\left(0\right)}$ is unavailable. Nevertheless, as the result of a procedure with a perfect first estimation step, it allows a comparison to quantify the performance of the second step.

For each parameter set and for each BgC method $\hat{S}={\hat{S}}^{\left(i\right)}$, *i*=0,…,5 we compute the Mean Absolute Deviation (MAD):

$$\text{MAD}(\hat{S})=\frac{1}{N}\overset{N}{\underset{\ell =1}{\sum }}\left(\frac{1}{n_{\text{reg}}}\overset{n_{\text{reg}}}{\underset{j=1}{\sum }}\left|\hat{S}(X_{j}^{\ell }|{\hat{\Theta }}_{\ell })-S_{j}^{\ell }\right|\right)$$

where ${\hat{\Theta }}_{\ell }=({\hat{\mu }}_{\ell },{\hat{\sigma }}_{\ell },{\hat{k}}_{\ell },{\hat{\theta }}_{\ell })$ denotes for each simulated array *ℓ*the estimated parameters corresponding to the used methods with the following conventions: 1/ ${\hat{\Theta }}_{\ell }$ is the true parameters for *i*=0; 2/ ${\hat{k}}_{\ell }=1$ for *i*=2,…,4 corresponding to the exponential distribution; 3/ ${\hat{\Theta }}_{\ell }$ represents the median over **X**^0,*ℓ*^for *i*=5. Simulation results are summarized in Table 4 for the parameter sets 1-6. The five first columns correspond to the excess risk ratio:

(10) $$\;R(i)=\text{MAD}({\hat{S}}^{\left(i\right)})/\text{MAD}({\hat{S}}^{\left(0\right)}),\;\text{for}\;i=1,\ldots ,5$$

and the last column indicates the reference risk $\text{MAD}({\hat{S}}^{\left(0\right)})$.

**Table 4:** Excess risk ratio of background corrected raw-scale intensities

***μ*,*σ*,*k*,*θ***	**R(1)**	**R(2)**	**R(3)**	**R(4)**	**R(5)**	**MAD**$\left({\hat{S}}^{\left(0\right)}\right)$
set 1	1.00	4.16	1.77	1.52	1.16	2.34
set 2	1.00	4.10	1.90	1.66	1.20	11.7
set 3	1.00	1.00	4.69	1.00	1.00	4.57
set 4	1.00	1.00	3.71	1.00	1.02	31.4
set 5	1.00	1.00	2.11	1.00	1.15	2.95
set 6	1.00	1.00	1.46	1.00	1.35	17.2

Mean Absolute Deviation (MAD) of the background corrected intensities for methods ${\hat{S}}^{\left(j\right)}$, *j*=1,…,5 divided by the MAD for the theoretical normal-gamma BgC with the true parameters (method ${\hat{S}}^{\left(0\right)}$), from the simulation data set (*S*_1_). Column 1: normal-gamma, column 2: normexp-MLE, column 3: normexp-RMA, column 4: normexp-NP, column 5: background subtraction. The MAD of the theoretical normal-gamma deconvolution with the true parameters is given as reference in column 6.

The normal-gamma BgC provides the same quality when the parameters are known or estimated. This holds when the data are generated either from a normal-gamma or a normexp model. Normexp-NP shows good behaviors when the data come from a normexp model but has a risk increase of order 60% if the data come from a normal-gamma model. Normexp-MLE provides good results for normal-exponential data but fails when the data come from a normal-gamma model. Not surprisingly, as already pointed by Xie *et al*[8], normexp-RMA has a poor behavior. The background subtraction method with a maximal quality loss of 32% offers an acceptable alternative in terms of risk. Indeed, most of the intensities being small, putting them to 0 does not affect significantly the MAD value. Let us recall, however, that from a practical point of view, the major disadvantage of background subtraction is the elimination of a considerable number of probes.

In practical experiments, the data are usually transformed before the analysis. To address this issue, the MAD is computed on log-transformed intensities (see Additional file 1: Section 4 for details), and the excess risk ratio is displayed in Table 5. The normal-gamma BgC presents a smaller error of estimation than the methods based on the normexp model. The MAD from normexp-MLE generates the highest value, and normexp-RMA and normexp-NP show a a similar moderate excess risk. Nevertheless the differences between the BgC methods are less pronounced than the one observed at the raw scale. However, with an excess risk ratio between 16% and 33%, the normexp methods notably under perform the BgC based on the normal-gamma model in terms of signal estimation.

**Table 5:** Excess risk ratio of background corrected log-transformed intensities

***μ*,*σ*,*k*,*θ***	**R(1)**	**R(2)**	**R(3)**	**R(4)**
set 1	1.00	1.32	1.18	1.17
set 2	1.00	1.28	1.16	1.16
set 3	1.00	1.00	2.98	1.00
set 4	1.00	1.00	2.45	1.00
set 5	1.00	1.00	1.81	1.00
set 6	1.00	1.00	1.39	1.00

Mean Absolute Deviation (MAD) of the background corrected intensities for methods ${\hat{S}}^{\left(j\right)}$, *j*=1,…,5 divided by the MAD for the theoretical normal-gamma BgC with the true parameters (method ${\hat{S}}^{\left(0\right)}$), from the simulation data set (*S*_1_). Column 1: normal-gamma, column 2: normexp-MLE, column 3: normexp-RMA, column 4: normexp-NP.

The MAD computation offers a global comparison of the various BgC methods in terms of signal estimation. We refine this analysis by examining the absolute deviation (AD) of the estimated signal for each signal intensity at the raw and log scales, respectively defined as:

$$\mathrm{AD}(S_{j})=\frac{1}{N}\overset{N}{\underset{\ell =1}{\sum }}\left|\hat{S}(X_{j}^{\ell }|{\hat{\Theta }}_{\ell })-S_{j}^{\ell }\right|$$

$$\mathrm{AD}(\log(S_{j}))=\frac{1}{N}\overset{N}{\underset{\ell =1}{\sum }}\left|\log\left(\hat{S}(\overset{\ell }{\underset{j}{X}}|{\hat{\Theta }}_{\ell })\right)-\log\left(\overset{\ell }{\underset{j}{S}}\right)\right|$$

**Figure 3:**
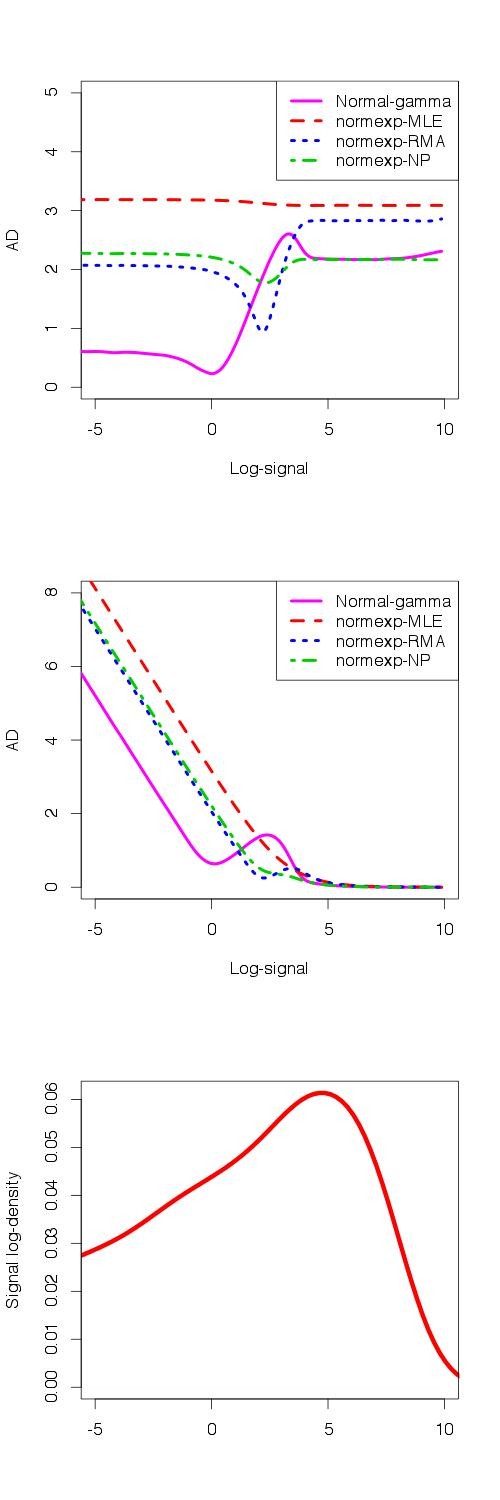
**Absolute deviation of the signal estimation on simulated data.** Logarithm of the Absolute Deviation of estimated signal on raw scale (first row), Absolute Deviation of log-transformed estimated signal (second row) and signal log-density (third row). Normal-gamma BgC (purple) and normexp BgC with MLE (pink), RMA (blue) and NP (green) parameters.

The first row of Figure 3 displays the logarithm of the AD at the raw scale, as a function of the log-signal intensity. We observe that normexp-MLE presents a larger AD for all values of the signal. On small intensities, the normal-gamma BgC outperforms the other methods, whereas normexp-RMA and normexp-NP present a smaller deviation on moderate intensities. For high values of the signal, normal-gamma shows a smaller error of estimation together with normexp-NP.

The absolute deviation on log-transformed intensities is presented on the second row of Figure 3. The normal-gamma BgC still presents the smallest error of estimation on weak intensities, but is outperformed by the other methods on moderate intensities. The four methods present similar AD values on high intensities. Besides, we observe than the error of estimation for all methods increases as the signal become weaker.

#### Robustness

In Section “Previous modelings”, we have underlined the slightly heavier right tail of the negative probe distribution. To ensure that the estimation remains acceptable under the assumption of an imperfect noise parametrisation, we compare the robustness of the normal-gamma method with normexp-NP, stated as the most robust by [8] and which we found competitive (see Section “Model-based background correction”). The errors of estimation computed from the simulation data set (*S*_2_) are presented in Additional file 1: Section 5. Both methods are robust with respect to non-normal noise distribution, and the normal-gamma BgC still offers a better quality of estimation than normexp-NP when the noise distribution departs from normality.

In conclusion, the normal-gamma background correction globally offers a better quality in signal estimation with respect to the normexp methods. Nevertheless, this improvement depends on the scale considered and does not steadily hold over the range of intensities.

### Operating characteristics

**Figure 4:**
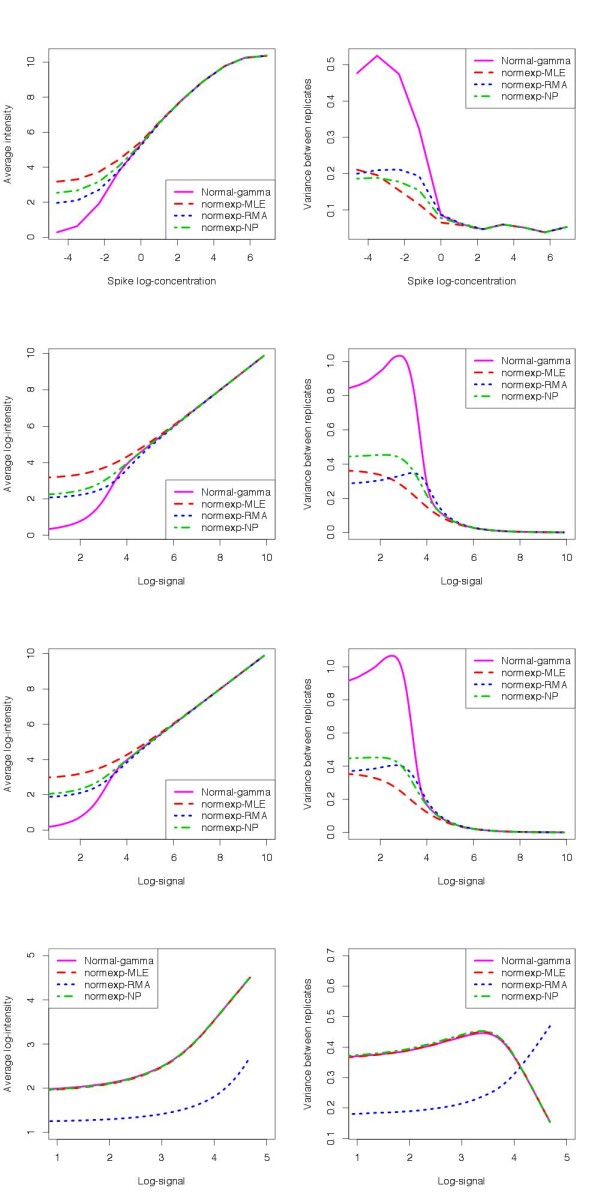
**Operating characteristics of the BgC methods on spike-in and simulated data.** Row 1: average spike intensities (left) and standard deviation of spike replicates (right) for all non-zero spike concentrations. Row 2 to 4: average intensity (left) and standard deviation of replicates (right) as a function of signal intensity. Row 2: normal-gamma simulation in data set (*S*_3_) (parameter set 7); Row 3: gamma signal and empirical background noise distribution (data set (*S*_4_)); Row 4 normal-exponential simulation in data set (*S*_3_) (parameter set 9).

Beyond the quality of estimation of the signal, the performance of a BgC procedure in practical experiments depends on its characteristics in terms of bias and variance. In this section, we compare the operating characteristics of the normal-gamma and normexp BgC both on simulated and spike-in data. The results are gathered in Figure 4. The background subtraction leading to probe deletion is not further considered. The data from (*E*_3_) are background corrected with the methods 1 to 4 described in Section “Background corrected intensity”. Quantile normalization based on both regular and negative probe intensities is applied, followed by log-transformation. The same procedures are implemented on the simulation data sets (*S*_3_) and (*S*_4_).

#### Bias-precision trade-off

The quality of a pre-processing method in microarray experiments can be characterised by its ability to distinguish between distinct values of the signal. Most of the procedures underestimate the signal fold-changes. This bias in fold-change estimation, called compression, has a negative impact on differential analysis. But the efficiency of a pre-processing method also depends on its precision, characterised by the variations of the corrected intensity for a given value of the signal. The trade-off between bias and precision is an indicator of the performance of a procedure. This issue can be understood by the example of a t-test statistic for a given probe differentially expressed between two groups: an important compression attenuates the difference of average intensities between the two groups, whereas a poor precision generates a high variance term in the denominator, which reduces the value of the test statistic.

The compression and precision obtained with the four BgC methods on the data set (*E*_3_) are presented on the first row of Figure 4. The first column displays the average intensity over the 34 spike bead types for each spike concentration. A similar saturation effect is observed for large concentrations with the four methods: for concentrations larger than 100pm, the relationship between log-intensity and log-concentration is not linear. Moreover, as the concentration decreases, a compression of the signal is observed with all the methods, but is significantly less pronounced with the normal-gamma BgC.

The second column presents the average standard deviation between replicates over all spike bead types. We observe that the improvement in bias brought by the normal-gamma model is at the cost of a poorer precision. More generally, the precision increases with the compression for the four methods.

#### Innate offset

Shi *et al*[10] highlight the role played by the ”innate offset”, defined as the typical intensity assigned to the non-expressed genes by a pre-processing procedure, in the unbalanced bias-variance trade-off of the various BgC methods: the strategies which show the smallest innate offset usually generate less bias but present a poorer precision. On spike-in data sets, the innate offset is defined as the mean of the intensities measured on spike probes with concentration zero. The results displayed in Table 6 confirm the observations from Shi *et al*[10]: as underlined above, the normal-gamma BgC exhibits the smallest precision associated with the smallest bias with a slope from the linear regression of intensities on log-concentrations close to 1. The largest offset combined with the highest precision and the largest bias are observed for normexp-MLE. 

**Table 6:** Innate offset and operating characteristics

**BgC**	**Innate offset**	**Stand. Dev.**	**Slope**
normexp MLE	23.4	0.095	0.74
normexp NP	12.4	0.100	0.80
normexp RMA	6.9	0.110	0.86
normal-gamma	1.5	0.200	0.99

Innate offset, average standard deviation of spike replicates and slope of the linear regression of the spike average intensity on the log-concentration.

Shi *et al*[10] propose to compare the pre-processing methods in a more equal way by adding an offset to the background-corrected quantile-normalised intensities before log-transformation, in order to align the innate offsets of the various pre-processing strategies. Our results presented in Additional file 1: Section 6.1, indicate that the characteristics between the four BgC present more similarity after equalizing the innate offsets, but a slight difference remains between normexp-MLE and the other BgC methods on small intensities. Nevertheless, prior to the offset equalisation, the methods studied in this paper do not present the large range of bias-precision trade-offs observed in the pre-processing strategies considered in [10]. In this context, the equalisation of the innate offsets does not appear sufficient to completely erase the differences between BgC methods.

#### Operating characteristics on simulated data

In order to reinforce the validation of the normal-gamma parametrisation for the noise-signal distribution, we compare the operating characteristics obtained on spike-in data to the ones provided by the normal-gamma simulated data from set (*S*_3_). The spike concentration, used as references to assess the bias and precision of the procedures on spike-in data, is replaced by the true value of the signal. The results are displayed on the second row of Figure 4. The first column presents the average intensity as a function of the signal log-intensity, and the standard deviation of the replicates is shown in the second column. The trends are very similar to the ones observed on spike-in data, with a small difference for the variance with normexp-RMA. Besides, we observe that the compression in small intensities generated by the four BgC methods is purely a statistical effect. However, the signal attenuation in high intensities observed on spike-in data is not present on simulated data. Indeed, it has already been suggested that this phenomenon could come from saturation in light intensity on microarrays.

Furthermore, we address the departure from normality observed on the negative probe distribution by simulating microarrays with a gamma distributed signal and a non-normal background noise (data set (*S*_4_)). In order to get a realistic noise distribution, the background noise and the negative probe intensities are sampled from the quantile-normalised negative probe intensities from all arrays in (*E*_3_) (see details in Additional file 1: Section 2.2). The operating characteristics of the four BgC methods are presented on the third row of Figure 4. We observe that the slight difference between normal-gamma simulations and spike-in data with normexp-RMA, observed on the second row of Figure 4, is partially corrected by generating a non-normal background noise.

The same quantities are computed based on normal-exponential simulated data with parameter sets 8 and 9. The results are displayed on the fourth row of Figure 4 for set 9, and on Figure G in Additional file 1 for set 8. The comparison between the operating characteristics of the four BgC methods are absolutely not consistent with the observations from the spike-in data. In particular, the normal-exponential simulated data provide almost identical bias and precision curves for normal-gamma, normexp-NP and normexp-MLE, whereas these methods exhibit notable differences on spike-in data.

The parallel drawn between the operating characteristics of the four BgC methods on spike-in and simulated data confirms that the gamma model represents a much more accurate parametrisation for the signal distribution than the usual exponential model.

### Differential expression analysis

The BgC methods are compared from a practical point of view through a differential expression analysis performed on the dilution data set (*E*_4_), based on the hierarchical linear model approach from Smyth [21] implemented in the limma package. This procedure provides p-values from a moderated t-statistic. A first analysis is run on the two pure samples (proportions 100%/0% and 0%/100%) to define the ”true” differentially expressed (DE) and non-differentially expressed probes. A second differential expression analysis performed on the two mixed samples (proportion 75%/25% and 25%/75%) is used to assess the performance of the BgC methods. The moderated t-test statistic implemented in the limma package includes a variance term representing the variation of the gene intensity across all arrays, as well as hyperparameters computed from the whole data set intensities. Therefore, in order to get independent results, the two analyses are performed on separate linear models.

The estimate proportion of DE probes in pure samples computed with a convex decreasing density procedure [22] is 28% for all methods. Thus, in order to be conservative, we define the probes with the 20% smallest p-values as ”true DE”, and the probes with the 40% highest p-values as ”true non-DE”. Moderated t-statistic values are then computed from the comparison of the two mixed samples. The p-values from true DE and non-DE probes are ordered. The area under the ROC curve (AUC) is used to quantify the sensitivity of each BgC method, the largest value of the AUC corresponding to the highest sensitivity. The four methods present similar AUC values but the normal-gamma BgC is slightly less competitive.

**Figure 5:**
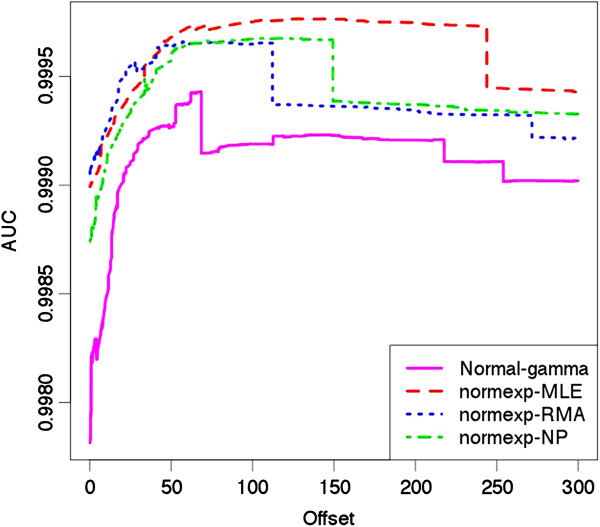
**AUC as a function of added offset.** AUC from moderated t-test for mixed sample differential analysis in data set (*E*_4_) (proportion 25%/75% and 75%/25%) for different values of offset.

A similar analysis is run with the addition of an offset prior to log-transformation. Figure 5 displays the AUC values as a function of the added offset with each BgC method. The values observed for an offset equal to zero correspond to the sensitivity when a simple log-transformation is applied. As already highlighted by Shi *et al*[10], we observe that the addition of a moderate offset increases the sensitivity of all BgC methods. For any value of the offset, the normexp methods outperform the normal-gamma. The results obtained with the different normexp BgC methods are very similar, but we note that the highest sensitivity is achieved with normexp-RMA for offsets smaller than 50, and with normexp-MLE for offsets larger than 50. 

The BgC methods can also be compared regarding their ability to order a set of measured intensities corresponding to increasing or decreasing probe concentrations. This framework can refer, for example, to a longitudinal study where the gene expression is repeatedly measured at different times. The correlation between the mixture proportion and the intensity is analysed on the dilution data set (*E*_5_). For the true DE probes, the intensity is expected to be increasing or decreasing with the proportion.

The dilution data sets (*E*_4_) and (*E*_5_) are based on the same pure biological samples. Therefore, true DE and non-DE probes defined on (*E*_4_) can be considered in the analysis of the data from (*E*_5_). The BeadChips used in experiments (*E*_4_) and (*E*_5_) are different, but some bead types are present on both devices. By mapping the annotation files from both BeadChips, the sets of probes respectively defined as DE and non-DE on (*E*_4_), and present on (*E*_5_) are extracted.

For each probe, the Spearman correlation coefficient is computed between the vector of mixture proportions and the observed intensities. This provides a test statistitic based on the ranking of the background corrected intensities, which allows a comparison of the BgC methods independently from the scale at which the data are analysed, provided that the transformation applied to the data is increasing. In particular, the results are not affected by the addition of an offset. The correlation coefficient is computed separately on microarrays with starting RNA quantities 250ng, 100ng, 50ng and 10ng. The coefficient is expected to be close to 1 in absolute values for the DE probes, and close to 0 for the non-DE. The probes are ranked according to their correlation coefficient value, and the resulting AUCs for each starting RNA quantity are displayed in Table 7. We observe that the normal-gamma BgC is slightly but steadily less sensitive that the other methods. The AUCs observed with the different normexp estimates are very similar and do not allow to assess the superiority of one method over the others.

**Table 7:** AUC from Spearman correlation test

	**Normal-**	**Normexp-**	**Normexp-**	**Normexp-**
	**gamma**	**MLE**	**RMA**	**NP**
250ng	0.9778	0.9812	0.9813	0.9820
100ng	0.9774	0.9807	0.9809	0.9808
50ng	0.9805	0.9834	0.9832	0.9841
10ng	0.9782	0.9818	0.9787	0.9816

AUC from Spearman correlation test between the proportion and the intensity in the dilution data set (*E*_5_), for the four BgC methods, and the four RNA starting concentrations.

## Conclusions

In many microarray experiments, background noise correction is an important issue in order to improve the measurement precision. Model-based background correction procedures have been developed as an alternative to the default background subtraction from Illumina BeadStudio which has proved to remove informative probes. The usual normal-exponential model considered for the noise-signal distribution has already been pointed out as inappropriate for Illumina BeadArrays [12]. Our observations confirm this result by highlighting the poor fitting of the normexp reconstructed densities on observed intensities with three different parameter estimates. We propose an alternative model based on a more flexible parametrisation of the signal which is assumed to follow a gamma distribution, as well as the associated background correction. The reconstructed density offers a better fit of the distribution of the observed intensities, validating this new model as more appropriate for Illumina microarrays. Moreover, the estimators based on the normal-gamma model are likely to apply to other microarray technologies including Affymetrix and single color Agilent, as an extension of the normexp model.

We compare the performance of the background correction procedures based on the normal-gamma and normal-exponential models on simulated and experimental data sets. Our simulation study indicates that the normal-gamma model brings an overall improvement in terms of signal estimation, characterised by a smaller average difference between the true signal and the background corrected intensity. But surprisingly, the differential expression analysis run on two dilution data sets shows that the improvement in terms of parametrisation does not have a positive impact on practical experiments, the normal-gamma correction exhibiting a slightly poorer sensitivity than the normexp methods. This result may be explained in two ways.

On one side, the operating characteristics of the background correction procedures are compared on a set of spike-in data, which allow to connect the probe intensity with the concentration of the target gene in the biological sample. We note that the normal-gamma model generates less bias than the normexp methods, but at the cost of a loss in precision. With the addition of an offset prior to the log-transformation, which provides balance in the bias-precision trade-off of the different methods, the operating characteristics appear similar, suggesting comparable performance.

On the other side, we examine the error in signal estimation as a function of the signal on log-scale simulated data. The normal-gamma model outperforms the other methods on small intensities, but is less competitive on moderate intensities. Due to the marked compression of the recovered intensity when the signal decreases, the improvement in terms of signal estimation for the small intensities has a weak effect on the differential expression analysis. Thus, the smaller average error of estimation observed with the normal-gamma background correction does not result in a higher sensitivity in practical experiments.

Besides, the parallel drawn between the operating characteristics of the different background corrections obtained, on the one hand with spike-in data and on the other hand with normal-gamma simulated data, highlights high similarities. The simulations from the normal-gamma model recover subtile differences between background correction procedures, whereas simulations from the normexp model totally fail to reproduce the trends observed on spike-in data. These considerations enhance the validation of the normal-gamma model for Illumina microarrays, and illustrate the potential of the normal-gamma simulations for the comparison of pre-processing procedures. Furthermore, the similarities between the observations from spike-in and simulated data are increased by sampling the background noise from the empirical negative probe distribution which suggests that an improvement in modeling could be brought by a non-normal parametrisation of the background noise.

In conclusion, this paper addresses the lack of fit of the usual normal-exponential model by proposing a more flexible parametrisation of the signal distribution as well as the associated background correction. This new model proves to be considerably more accurate for Illumina microarrays, but our results indicate that the improvement in terms of modeling does not lead to a higher sensitivity in differential analysis. Nevertheless, this realistic modeling makes way for future investigations, in particular to examine the characteristics of pre-processing strategies.

## Endnote

^a^http://www.bioconductor.org

## Competing interests

The authors declare that they have no competing interests.

## Authors’ contributions

The NOWAC data were provided by EL, Principal Investigator of TICE project. Statistical and computational aspects were developed by SP and YR. All authors read and approved the final manuscript.

## Supplementary Material

Additional file 1 Supplementary Material 1 provides a description of the simulations, computing details and additional figures. Click here for file

Additional file 2 Supplementary Material 2 gathers the scripts used to produce the tables and figures. Click here for file

Additional file 3 **Supplementary Material 3 is a zip file which contains three text files with the observed intensities of the ten microarrays from data set (*E***_**1**_**).** Click here for file

## Fundings

Grant: ERC-2008-AdG 232997-TICE “Transcriptomics in cancer epidemiology”.
