# Supplementary material for: Generalization of the normal-exponential model: exploration of a more accurate parametrisation for the signal distribution on Illumina BeadArrays
Source: BMC Bioinformatics. 2012 Dec 11;13:329. doi: 10.1186/1471-2105-13-329 (PMC3599453; doi:10.1186/1471-2105-13-329)
Supplement: Additional file 1 — Supplementary Material 1 provides a description of the simulations, computing details and additional figures. [file 1471-2105-13-329-S1.pdf]

Supplementary Material 1

1 Non-parametric estimators of the signal

Wang and Wang (2011) developed a kernel-based deconvolution procedure implemented in the R-package `decon` to estimate a density from a sample measured with gaussian errors. The function`DeconPdf` provides a non-parametric estimator of the density up to a shift due to non-centered errors. Three bandwidth estimates are available, including a rule of thumbs estimate as well as two estimates computed by bootstrap-type methods. We have applied this procedure to the microarrays from data sets (E_1) and (E_2) presented in the article (Section 2.1). Figure A displays the non-parametric estimates of the signal density (black line) for one mice and one human microarray. Exponential and gamma densities are fitted to the decreasing part of the non-parametric estimate. We observe that the gamma density offers a better fit than the exponential, which fails to handle the heavy right tail.

However, it is important to note that kernel-based procedures which produce a smooth estimator does not offer a good reconstruction on intervals where the density is highly irregular. Smaller bandwidths recover better sharp variations of the density, but produce less stable estimates. We illustrate this phenomenon on simulated data with gamma distributed signal and gaussian noise. The estimators of the signal density computed with `DeconPdf` using the three data-driven bandwidth estimates are displayed in Figure B, as well as the true density shifted from the noise mean. The largest bandwidth produces a stable estimator which offers a very good fit of the right tail of the distribution, but dramatically fails around 0. As the bandwidth decreases, the estimator is sharper in 0 but at the price of instabilities. Thus, non-parametric estimation procedures based on kernel smoothing allow to explore the potentiality of the gamma parametrization to fit the right tail of the signal density, but may not be indicative of the signal distribution around 0.

In conclusion, the non-parametric estimate of the signal density exhibits heavy right-tail that can not be fitted by an exponential distribution density, calling for a more flexible model. Nevertheless, the instability of the non-parametric estimator, as well as the smoothing operated on the breakpoint limits the interpretation for small intensities.

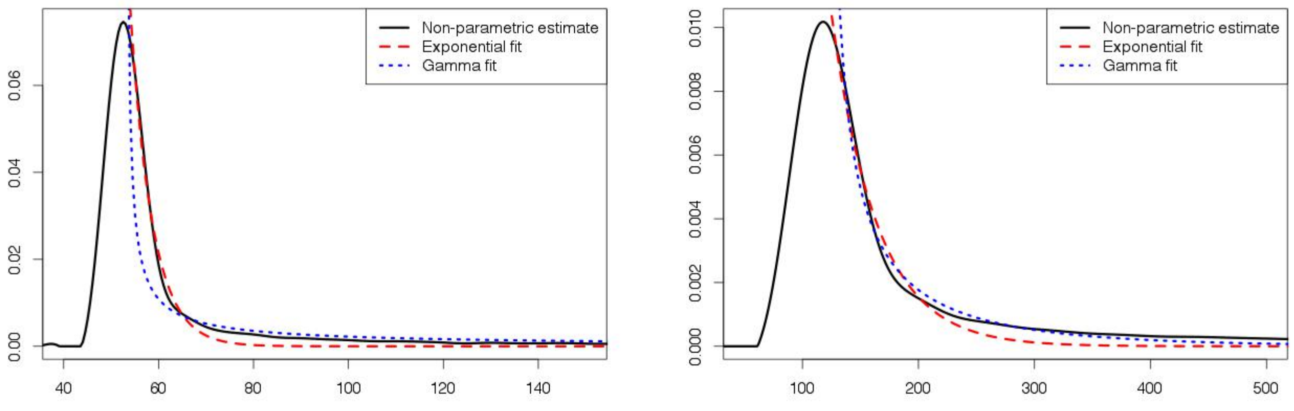

Figure A. Non-parametric estimate of the signal density, and exponential and gamma densities fitted on the decreasing part of the estimate, for a human (left) and a mouse (right) microarray.

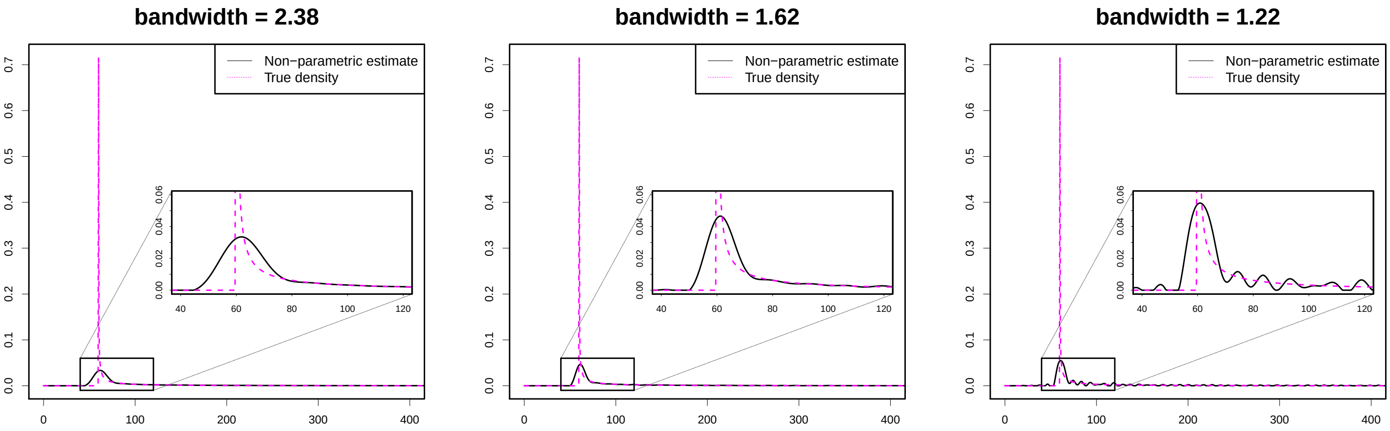

Figure B. Kernel-based deconvolution estimators of the signal density from normal-gamma simulated data, computed with the R package `decon` using the three data-driven bandwidth estimate: `dnrd` (left), `dboot2` (center), `dboot1` (right). The frame on each graph presents a zoom on the values $x \in [40, 120]$ and $y \in [0, 0.06]$.

2 Experimental and simulated data sets

2.1 Laboratory methods for NOWAC data set (E_1)

Data set (E_1) consists of the gene expression profiles in peripheral blood of ten controls from the Norwegian Women And Cancer cohort. The gene expression profiles were measured on source cells using Illumina Human HT-6 v4 Expression BeadChip (Illumina, San Diego, CA), which enables genome-wide expression analysis (more than 48 000 transcripts) of six samples in parallel on a single microarray. A restricted set of more than 25,000 probes is also considered by removing non-reliable probes according to Illumina's annotation files. The Illumina TotalPrep RNA amplification Kit (Ambion Inc., Austin, TX) was used to amplify RNA for hybridization on Illumina BeadChips. To synthesize the first strand cDNA by reverse transcription, we used totalRNA from each sample collected above. Following the second strand cDNA synthesis and cDNA purification step, the in vitro transcription to synthesize cRNA was prepared overnight within 12 hours. The microarray service was provided by NMC-NTNU, a Norwegian national technology platform supported by the functional genomics program (FUGE) of the Research Council of Norway.

2.2 Simulation of microarray data

Simulation experiment (S_1)**.** Microarray data are simulated from both normal-gamma and normexp models. In order to get realistic values of the parameters, six sets of parameters are computed by applying normal-gamma MLE, as well as normexp MLE and RMA estimation methods to observed intensities from one microarray from (E_1) and (E_2). The values of the six sets of parameters are summarized in Table A (sets 1-6). For each set of parameters, $N = 100$ random arrays are generated under the normal-gamma model, with $n_{\text{reg}} = 25000$ regular probes and $n_{\text{neg}} = 1000$ negative probes. For each repetition $\ell = 1, \ldots, N$, two independent samples $\mathbf{X}^\ell$ and $\mathbf{X}^{0,\ell}$, corresponding respectively to the expression levels of the regular and negative probes, are generated:

- $\mathbf{X}^\ell = \{X_j^\ell = S_j^\ell + B_j^\ell, j = 1, \ldots, n_{\text{reg}}\}$ with

  $\{S_j^\ell, j = 1, \ldots, n_{\text{reg}}\}$ independent identically distributed (i.i.d.) sample from a gamma distribution with shape k and scale θ.

  $\{B_j^\ell, j = 1, \ldots, n_{\text{reg}}\}$ i.i.d. sample from a normal distribution with mean μ and variance σ^2.

- $\mathbf{X}^{0,\ell} = \{B_j^{0,\ell}, j = 1, \ldots, n_{\text{neg}}\}$ i.i.d. sample from normal distribution with mean μ and variance σ^2.

Simulation experiment (S_2)**.** The procedure is similar to (S_1), but the background noise and the negative probe intensities are generated from a mixture distribution

$$(1-p)\mathcal{N}(\mu\sigma) + p\chi^2(3, 55)$$

where $\chi^2(3, 55)$ is a χ^2-distribution with 3 degrees of freedom and a non-centrality parameter equal to 55, for p in $(0.1, 0.25, 0.5, 0.75, 1)$. The values of the normal-gamma parameters are given in Table A, set 1.

Simulation experiment (S_3)**.** The parameter values for this experiment, computed from one array in experimental data set (E_3) are given in Table A: set 7 is computed from the normal-gamma model, and sets 8 and 9 are estimated from the normexp model with MLE and RMA parameter estimates. In order to mimic replicates from the same biological sample, the vector of signal intensities $\mathbf{S}$, drawn from a gamma distribution is identical on the N arrays. Then, for each repetition $\ell = 1, \ldots, N$, two independent samples $\mathbf{B}^\ell$ and $\mathbf{X}^{0,\ell}$ corresponding respectively to the intensities of the background noise on regular probes and the intensities of negative probes are generated.

- $\mathbf{X}^\ell = \{X_j^\ell = S_j + B_j^\ell, j = 1, \ldots, n_{\text{reg}}\}$ with

  $\{S_j, j = 1, \ldots, n_{\text{reg}}\} = \mathbf{S}$.

  $\{B_j^\ell, j = 1, \ldots, n_{\text{reg}}\}$ i.i.d. sample from a normal distribution with mean μ and variance σ^2.

- $\mathbf{X}^{0,\ell} = \{B_j^{0,\ell}, j = 1, \ldots, n_{\text{neg}}\}$ i.i.d. sample from a normal distribution with mean μ and variance σ^2.

Finally, $\mathbf{X}^\ell$ and $\mathbf{X}^{0,\ell}$ stand for the regular and negative probe intensities on array ℓ. Note that the parameter sets 8 and 9 with a shape value of 1 correspond to an exponential distribution.

Simulation experiment (S_4)**.** This data set is simulated based on a gamma distributed signal and a non-normal background noise. In order to get a realistic distribution of the background noise, the negative probe intensities from the 48 arrays in experimental data-set (E_3) are quantile-normalized independently from the regular probes, and gathered in a vector $\mathbf{D}_{\text{neg}}$. The signal identical over all arrays is simulated from a gamma distribution with parameters k and θ from set 7. Then, for each array ℓ, two vectors B^ℓ and $\mathbf{X}^{0,\ell}$ of length n_{reg} and n_{neg} standing for the background noise and the negative probe intensities are sampled with replacement from $\mathbf{D}_{\text{neg}}$.

	μ	σ	k	θ	Exp. data set	est. method
set 1	53	4.4	0.12	1785	(E_1)	f^{ng} + MLE
set 2	138	24	0.11	4949	(E_2)	f^{ng} + MLE
set 3	43.5	5.8	1	226	(E_1)	f^{nexp} + MLE
set 4	170	41	1	505	(E_2)	f^{nexp} + MLE
set 5	52.8	5.0	1	8.33	(E_1)	f^{nexp} + RMA
set 6	223	37	1	33.8	(E_2)	f^{nexp} + RMA
set 7	93	11	0.08	3230	(E_3)	f^{ng} + MLE
set 8	69	13	1	277	(E_3)	f^{nexp} + MLE
set 9	92	14	1	10.5	(E_3)	f^{nexp} + RMA

Table A. The nine sets of parameters used in the simulations by using on array (E_1), (E_2) and (E_3) with the three methods of estimation. The $k = 1$ value corresponds to a normexp model with an exponential distribution with mean θ.

3 Fit of normexp and normal-gamma model on experimental data sets (E_1) and (E_2)

3.1 Fit of normal model on negative probe distribution

On several arrays from (E_1) and (E_2), we have compared the density histogram of negative probes to the *plug-in* normal density $f^{\text{norm}}_{\hat{\mu},\hat{\sigma}}$ obtained by using robust estimators of the parameters (μ, σ):

$$\begin{cases} \widehat{\mu} = \text{median}(\{X_j, j \in J_0\}) \\ \widehat{\sigma} = \text{median}(\{|X_j - \widehat{\mu}|, j \in J_0\})/0.6745. \end{cases}$$

The results of this comparison, similar through the arrays, are illustrated in Figure C on one array from (E_1) and (E_2). This figure presents a regular density histogram of the negative probe intensities as well as the *plug-in* normal density. The empirical distribution is essentially normal but we notice a slightly heavier right tail.

3.2 Fit of the normexp and normal-gamma models.

Figure D presents the fit of the normal-gamma and normexp models with various parameter estimates on two arrays from (E_1) and (E_2).

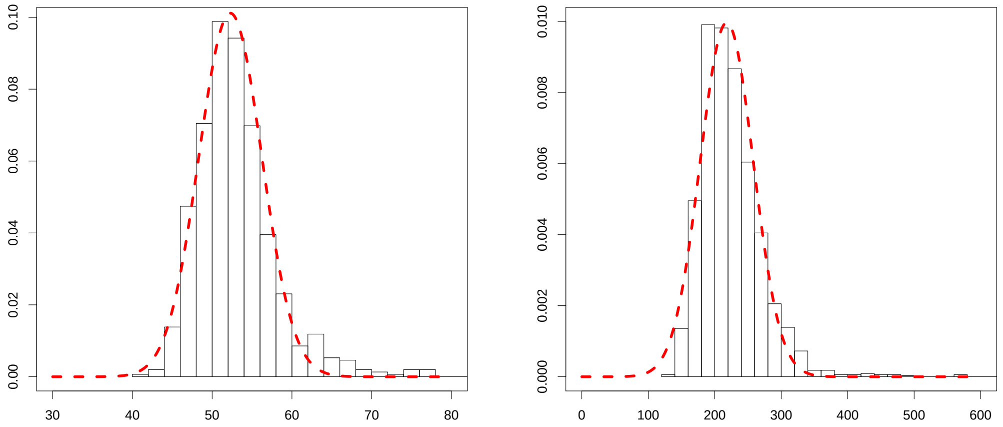

Figure C. Regular density histogram of the negative probe intensities and *plug-in* normal density using a robust estimate of the parameters (red dotted line) built on the negative probes. Left: one array from (E_1); right: one array from (E_2).

4 Quality of the signal estimation at the log-scale on the data set (S_3) (set 7)

For each background correction procedure $\widehat{S}^{(i)}$ $i = 0, \dots, 4$ described in Section 6.2.4, we compute the Mean Absolute Deviation of the bakground corrected log-transformed intensities over $N = 100$ repetitions. Note that the background corrected values after background subtraction include negative values and therefore can not be log-transformed. For each parameter set 1 to 6 and for each BgC methods $\widehat{S} = \widehat{S}^{(i)}$, $i = 0, \dots, 5$ the MAD on the log-transformed intensities is defined as:

$$\mathrm{MAD}(\log(\widehat{S})) = \frac{1}{N}\sum_{\ell=1}^{N}\left(\frac{1}{n_{\mathrm{reg}}}\sum_{j=1}^{n_{\mathrm{reg}}}\left|\log(\widehat{S}(X_j^\ell|\widehat{\Theta}_\ell)) - \log(S_j^\ell)\right|\right)$$

For each method $\widehat{S}^{(i)}$, we compute the excess risk ratio:

$$R(i) = \mathrm{MAD}(\widehat{\log}(S^{(i)}))/\mathrm{MAD}(\widehat{\log}(S^{(0)})), \quad \text{for } i = 1, \dots, 5$$

The results are displayed in Table 5.

5 Robustness with respect to non-normal background noise.

We check the robustness of the normal-gamma BgC with respect to heavier right tails in the negative probe distribution from the simulation data set (S_2), and compare the results obtained with normexp-NP. Figure E presents the density of the mixture distribution used for checking robustness. Table B presents the MAD computed from the normal-gamma BgC (first column) and the normexp BgC with NP estimation (second column).

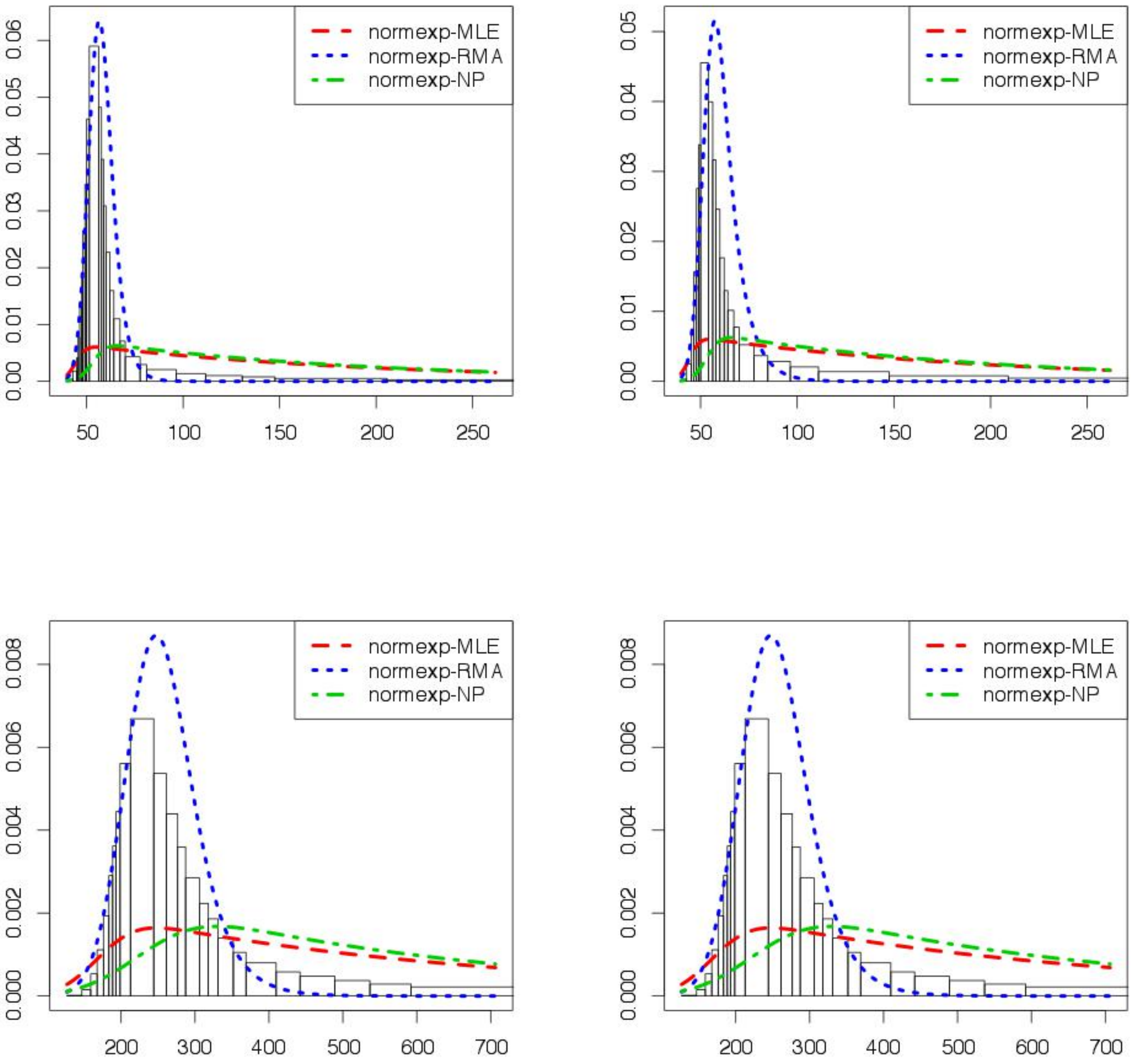

Figure D. Normal-gamma and normal-exponential fit on one array from (E_1) with the full set of regular probes (first row) and one array from (E_2) (second row). On each figure the irregular density histogram of the regular probe intensities is displayed. On left column are presented the plug-in normexp density with MLE, RMA and NP parameter estimates, and on the right column the plug-in normal-gamma density estimator, together with the normexp-RMA estimator.

p	$\text{MAD}(\widehat{S}^{(1)})$	$\text{MAD}(\widehat{S}^{(4)})$
0	2.38	3.59
0.10	3.12	4.88
0.25	4.19	6.51
0.50	5.70	8.72
0.75	6.82	10.53
1	7.59	12.01

Table B. MAD of background corrected intensities from simulated data with background distribution defined as a mixture $(1-p)\mathcal{N}(50,4)+p\chi^2(3,55)$ and signal is generated from a $\Gamma(0.12,1785)$.

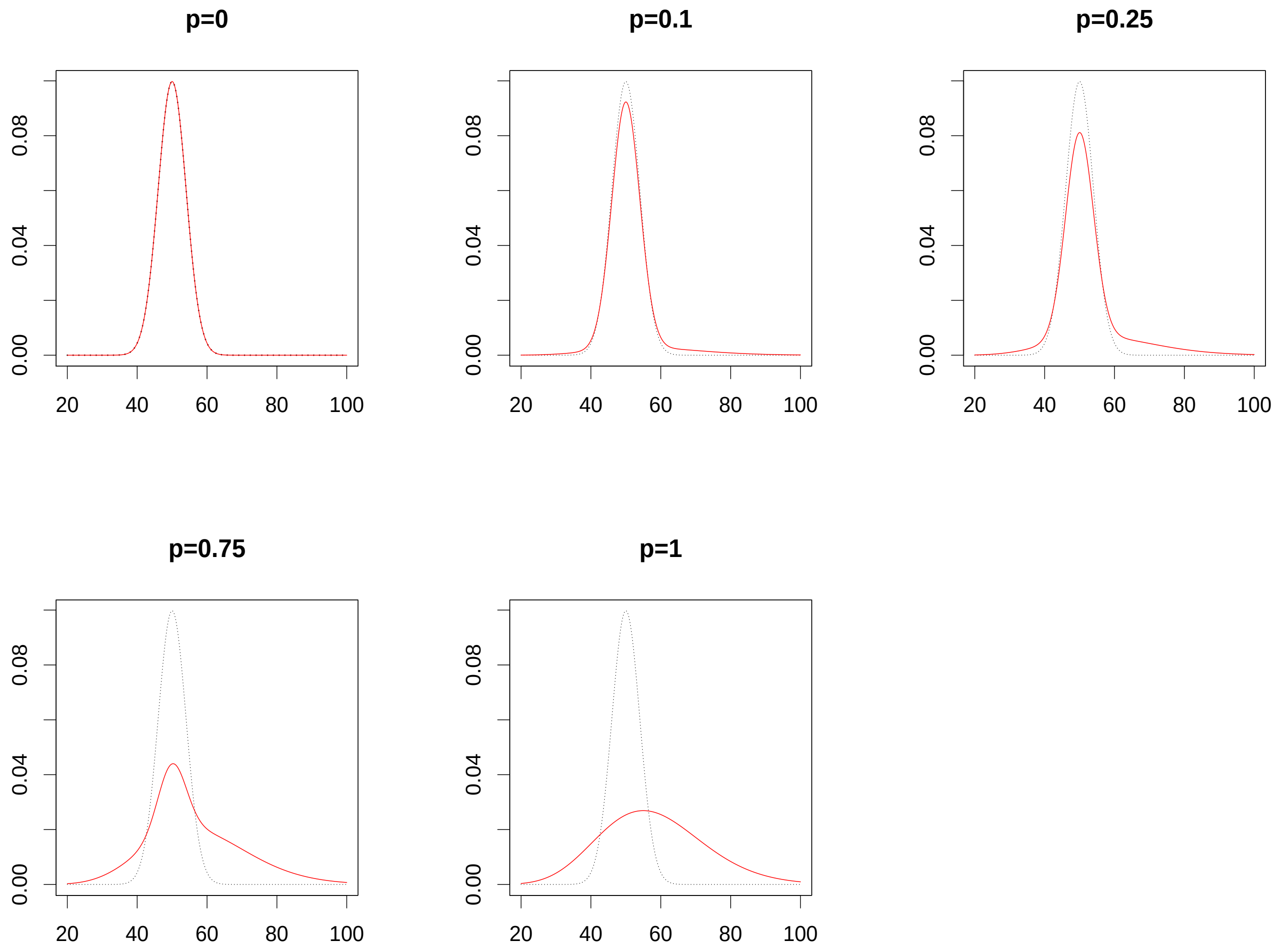

Figure E. Density of the mixture distribution $(1-p)\mathcal{N}(53,4.4)+p\chi^2(3,55)$ for various values of p.

6 Operating characteristics

6.1 Equal Innate offset on spike-in data set (E_3)

An offset has been added to the regular probe intensities prior to log-transformation for each method, in order to equalize innate offsets. The results displayed in Figure F corresponds to a total offset equal to 100 (i.e. an added offset of 98.5 for the normal-gamma BgC, 76.5 for normexp-MLE, 93 for normexp-RMA and 87.6 for normexp-NP). The first column presents the average intensity, the second column displays the estimated log-ratio for each pair of consecutive concentrations, corresponding to a true fold-change of 3 or 3.33.. The third column shows the standard deviation between replicates.

We observe that the precision is globally similar, but the difference on small concentration is enlightened by the observation of the fold-change for every pair of consecutive concentrations. We note that normexp-MLE provides a smaller bias (larger log-ratios) and a poorer precision.

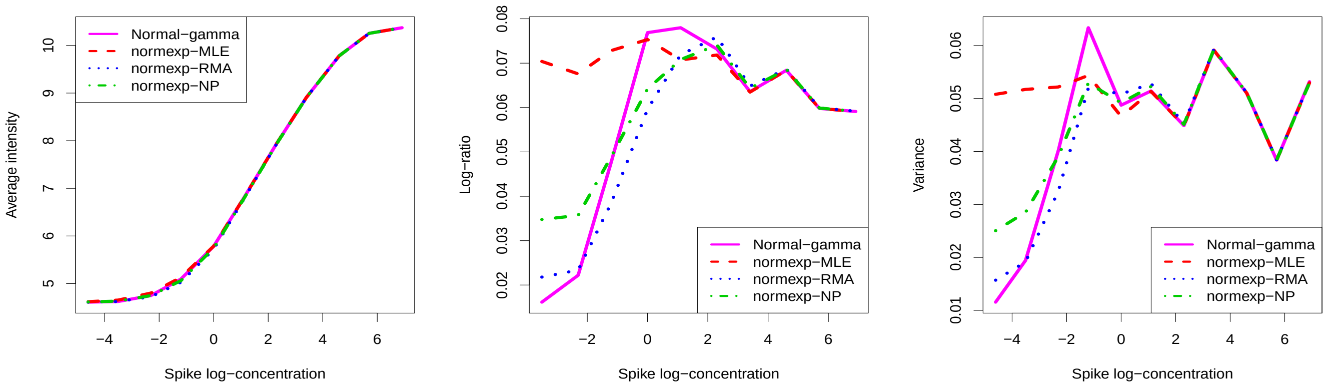

Figure F. Average spike intensity (left), average log-ratio for every pair of consecutive spike concentrations (center), and average standard deviation between spike replicates (right) as a function of the spike log-concentration after equalization of the innate offsets on raw data.

6.2 Operating characteristics on normal-exponential simulated data

The operating characteristics of the four BgC methods on the data set (S_3) with parameter set 8 are displayed in Figure G.

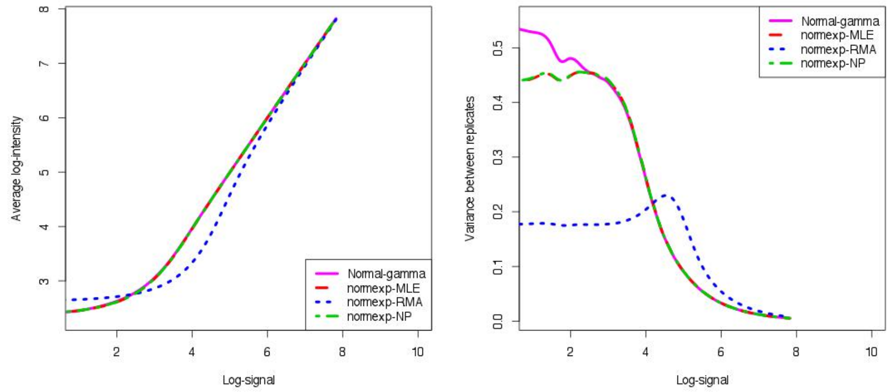

Figure G. Average log-intensities (left) and log-variance between replicates (right) for simulation data set (S_3) with parameter set 8.

7 Numerical implementation

7.1 Expression of the normal-gamma density

The convolution product of the normal and gamma density:

$$f^{\text{ng}}_{\mu,\sigma,k,\theta}(x) = \int f^{\text{gam}}_{k,\theta}(t) f^{\text{norm}}_{\mu,\sigma}(x-t)dt$$

does not have an analytic expression similar to the normal-exponential density. Nevertheless, an expression involving the Kummer's special mathematical function may be obtained by using the software `Mathematica`. Denoted by kum the M-Kummer's special function,

$$f^{\text{ng}}_{\mu,\sigma,k,\theta}(x) = 2^{1+k/2}\left(\frac{\sigma}{\theta}\right)^k f^{\text{norm}}_{\mu,\sigma}(x)\cdot$$
$$\left[\Gamma\left(\frac{k}{2}\right)\text{kum}\left(\frac{k}{2},\frac{1}{2},\frac{y^2}{2}\right) - \sqrt{2}y\Gamma\left(\frac{k+1}{2}\right)\text{kum}\left(\frac{k+1}{2},\frac{3}{2},\frac{y^2}{2}\right)\right]$$

where $y = (\sigma^2 + \theta(\mu - x))/(\sigma\theta)$. The R-package `fAsianOptions` implements the M-Kummer's special function. Unfortunately, it does not offer enough numerical stability to be used for microarray expressions, due to their range.

7.2 Computation of the normal-gamma density estimator by `fft`.

The normal gamma density $f_X = f^{\text{ng}}_{\mu,\sigma,k,\theta}$ is computed with the Fast Fourier Transform (`fft`) function on a regular grid of $[0,T]$ where $T = \mu + 5\sigma + q$ and q is the gamma distribution 0.99999-quantile, then interpolated on other points.

Approximation of f_X. Let φ_X denote the moment generating function of X,

$$\varphi_X(t) = \mathbb{E}\left[e^{itX}\right], \quad \forall t \in \mathbb{R}.$$

Then

$$\varphi_X(t) = \varphi_S(t)\varphi_B(t) = (1 - it\theta)^{-k} e^{i\mu t} e^{-\sigma^2 t^2/2}.$$

Moreover, for every $x \in \mathbb{R}$,

$$\begin{aligned} f_X(x) &= \int_{-\infty}^{\infty} \varphi_X(t) e^{-itx} dt \\ &\simeq \int_{-A}^{A} \varphi_X(t) e^{-itx} dt \\ &\simeq \frac{2A}{N} \sum_{j=0}^{N-1} \varphi_X\left(-A + \frac{2A}{N}(j-1)\right) \\ &\quad \exp\left(-itx\left(-A + \frac{2A}{N}(j-1)\right)\right) \quad \text{(Riemann sum)} \end{aligned}$$

for large A and N and small ratio A/N. Let us denote by $\overline{f}_X$ this approximate.

Computation using `fft`. The `fft` function of `R` is defined as follows. For a vector $\mathbf{V}$ of length N,

$$\texttt{fft}(\mathbf{V}) = \sum_{j=1}^{N} \mathbf{V}[j] \exp\left(-\frac{2i\pi}{N}(j-1)(k-1)\right).$$

Let

$$\mathbf{U} = \frac{\pi}{A}(0 : N-1)$$
$$\mathbf{V} = \varphi_X\left(-A + \frac{2A}{N}(0 : N-1)\right)$$

and

$$\mathbf{W} = \frac{A}{N\pi}\exp(iA\mathbf{U})\texttt{fft}(\mathbf{V})$$

then for $k = 0, \ldots, N-1$,

$$\begin{aligned}\mathbf{W}[k] &= \frac{A}{N\pi}\exp(i\pi(k-1))\sum_{j=1}^{N}\varphi_X\left(-A + \frac{2A}{N}(j-1)\right)\\ &\quad \exp\left(-\frac{2i\pi}{N}(j-1)(k-1)\right) = \overline{f}_X(\frac{k-1}{A}).\end{aligned}$$

7.3 Normal-gamma MLE computation.

The parameters (μ, σ, k, θ) of the normal-gamma model are estimated by likelihood maximization, ensured by the R-function `optimx` with the following initialization values.

$$\begin{aligned}\mu_0 &= \text{mean}(X_j, j \in J_0),\\ \sigma_0 &= IQR(X_j, j \in J_0)/1.349,\\ \theta_0 &= (\text{sd}(X_j, j \in J)^2 - \sigma_0^2)/(\text{mean}(X_j, j \in J) - \mu_0),\\ k_0 &= (\text{mean}(X_j, j \in J) - \mu_0)/\theta_0,\end{aligned}$$

where IQR designs the interquartile range and the new parametrization

$$(p_1, p_2, p_3, p_4) = (\mu, \sigma, k\theta, \theta\sqrt{k}),$$

in order to get homogenous parameters.

8 Infer the negative probe intensities from Illumina detection p-values

8.1 Algorithm

For a given array, let X and N be the vectors of regular and negative probe intensities, and P the vector of detection p-values. For each regular probe j with intensity X_j, the detection p-value P_j is equal to the proportion of negative probes with intensity larger than X_j:

$$P_j = \frac{1}{n_{\text{neg}}} Card\{k, N_k > X_j\}$$

where n_{neg} is the number of negative probes. Let Q be the vector of ordered unique values of P, and denote by $\ell(Q)$ its length. For every $k \in \{2, \ldots, \ell(Q)\}$, let:

$$x_k^1 = \max\{X_j, P_j = Q_k\}$$
$$x_k^2 = \min\{X_j, P_j = Q_{k+1}\}$$

then

$$Q_{k+1} - Q_k = \frac{k}{n_{\text{neg}}}$$

where d is the number of negative probes whose intensity lies in $[x_k^1, x_k^2]$. Therefore, for every $k \in \{2, \ldots, \ell(Q)\}$, we infer d negative probes with intensity equal to

$$\frac{1}{2}(x_k^1 + x_k^2).$$

Moreover, if $\min(Q) = 0$, we infer a negative probe with intensity $\max\{X_j, P_j = 0\}$ and if $\min(Q) = d_0/n_{\text{neg}}$, we infer d_0 probes with intensity equal to $\min(X)$.

8.2 Performances

For the ten human microarrays, we have computed the parameters 1/ with the true negative probe intensities, 2/ with the set of inferred negative probe intensities, for the normal-gamma model and for the normexp model with MLE and NP parameters. (As the RMA algorithm do not uses the negative probe intensities, this comparison is not relevant). Table C presents the relative error on parameters and on background corrected intensities.

Method	μ	σ	k	θ	$\widehat{S}$
Normal-gamma	2.9 E-5	5.2 E-4	7.9 E-4	4.2 E-4	6.3 E-4
Normexp-MLE	4.7 E-6	5.8 E-5	$\star$	7.2 E-4	2.1 E-5
Normexp-NP	1.4 E-5	1.8 E-4	$\star$	7.2 E-6	1.3 E-4

Table C. Relative error of estimation from replacing negative probe intensities by inferred values. Column 2-5: parameters of the model; Column 6: background corrected values.
